# X-Ray Attenuation Properties of Additive Manufacturing and 3D Printing Materials for Mimicking Tissues in Radiographic Phantoms Measured by CT from 70 to 140 kV: 2025 Update

**DOI:** 10.3390/biomimetics11030202

**Published:** 2026-03-10

**Authors:** Thomas Hofmann, Martin Buschmann, Peter Homolka

**Affiliations:** 1 Center for Medical Physics and Biomedical Engineering, Medical University of Vienna, 1090 Vienna, Austria; 2 Division of Medical Radiation Physics, Department of Radiation Oncology, Medical University of Vienna and University Hospital Vienna, 1090 Vienna, Austria; martin.buschmann@meduniwien.ac.at

**Keywords:** radiology phantoms, medical imaging, X-ray imaging, anthropomorphic phantoms, tissue equivalent materials, additive manufacturing, optimization in imaging, quality control

## Abstract

Background: Phantoms are essential in medical imaging, providing reproducible and quantitative means for system and protocol evaluation, image quality assessment, and dosimetry without patient exposure. Additive manufacturing enables rapid, accurate fabrication of phantoms ranging from simple geometries to complex anthropomorphic models. Ongoing developments in 3D printing technologies and polymer formulations have enhanced mechanical properties and printability, but also affect X-ray attenuation behaviour, necessitating an update with current materials to facilitate the choice of appropriate materials mimicking body tissues in radiographic phantoms. Methods: Attenuation properties of 27 photopolymer resins and 22 thermoplastic filaments (based on PLA, ABS, HIPS, PETG/PCTG, and PVB) were quantified using a clinical CT scanner at 70–140 kV to establish reference data for material selection. Results: At 120 kV, resins exhibited attenuation values between 124 and 384 Hounsfield Units (HU), and filaments ranged from −69 to 308 HU (PLA-based filaments: 160 to 241 HU, ABS: −32 to 43 HU, PETG/PCTG: 151 to 308 HU, and HIPS: −69 to −22 HU). Energy dependence of HU values is presented from 70 to 140 kV tube potential. Compared to the 2021 study, a wider selection of X-ray opacities is available. Regarding SLA/DLP printing, resins with higher attenuation were identified, and flexible resins that had provided a choice of low attenuation printing materials in the range of 60 to 90 HU at 120 kV tended to replicate attenuation properties closer to rigid photopolymers; i.e., HU values were slightly higher. In FDM filaments, a wide variation in different PLA-, ABS-, and HIPS-based filaments is found. In copolymers from the PET/PCTG/PETG family, very inhomogeneous X-ray attenuations are still found, as anticipated. Conclusions: The range of X-ray attenuation observed demonstrates that commercially available 3D printing materials can replicate clinically relevant tissues and tissue-equivalent contrasts. Furthermore, the available range of attenuations has increased, as has the finer gradation of these materials. These findings support the design of patient- and task-specific imaging phantoms for optimization of acquisition protocols, image quality evaluation, and radiation dose studies, as well as facilitate the selection of appropriate phantom materials.

## 1. Introduction

In medical imaging and radiation therapy with ionizing radiation, phantoms [1] are used for a variety of tasks and have become indispensable in both research and development and clinical practice [2,3]. They are defined as physical [2] or digital [4,5,6] models simulating patient anatomy and X-ray interactions with varying degrees of accuracy and fidelity, ranging from simple slab phantoms to accurate anatomical replications.

Phantoms need to adequately mimic relevant biological conditions to be useful for medical research and for the ongoing optimization and quality control of imaging systems and procedures applied. This includes reproducing tissue properties, anatomical structures, contrast relationships, and mimicking clinically realistic scenarios, e.g., in virtual clinical trials [7]. Achieving such biomimicry requires materials whose X-ray interaction properties closely resemble those of biological tissues, as well as phantom geometries that replicate structures found in healthy and diseased patients with sufficient accuracy.

To realistically replicate patient-like structures, 3D printing offers a powerful approach and is widely employed for this purpose. In this context, the present study focuses on the characterization of 3D-printed materials used for designs that replicate both the structural and radiological behaviour of biological tissues, thereby enabling (pseudo)anthropomorphic phantoms with intricate, biologically inspired features. Additive manufacturing, in combination with 3D printing, has had a profound impact on medical imaging research. This is mainly due to the ready adoption by the research community and rapid advances in affordable printers alongside a rapidly expanding selection of 3D printing materials, enabling complex projects that were previously barely feasible. Additive manufacturing and 3D printing enable (pseudo)anthropomorphic designs with intricate details, such as airway and vascular networks [8], tumour models [9,10,11,12], or distinct sections replicating different tissues or organs [13,14] featuring anatomically appropriate background structures, e.g., embedding test lesions within realistic backgrounds featuring appropriate anatomical noise [15]. These designs allow precise studies of lesion detection, protocol and contrast-to-noise optimization, detection of system limits under clinical-like conditions, from mammography phantoms [16,17] simulating glandular patterns [18,19,20] to CT phantoms testing and validating advanced features ranging from quantitative measurements to comparisons of iterative reconstruction algorithms [21,22,23] or the accuracy and performance of spectral/dual-energy applications [24,25].

Radiographic phantoms critically rely on materials that accurately reproduce the X-ray interactions of the tissues being mimicked within appropriate accuracy [26,27,28,29]. Commercial 3D printing materials—including FDM filaments and photocurable resins for SLA/DLP printers—typically exhibit incorrect energy dependence in X-ray attenuation and scattering due to the base polymers’ low effective atomic number (Z_eff_). This issue must be properly addressed, as polychromatic spectra across a range of tube potentials and combined with varying filtrations are used in imaging procedures. Matching X-ray interactions across clinical energy ranges is often difficult. Phantoms thus balance realism with practical possibilities [28], as no material perfectly replicates a tissue for all X-ray photon energies in the imaging modality of interest. This is further complicated by challenges such as batch variations, proprietary formulations that also may change due to continued product development, and effects from printing parameters, which compromise reproducibility, accuracy, and the validity of results. The selection of 3D printing materials for radiographic phantoms, therefore, requires precise knowledge of their X-ray interaction properties.

Cataloguing 3D printing materials for their X-ray attenuation across relevant diagnostic energy ranges is thus essential for material selection, with existing studies providing valuable data [27,30,31,32,33,34,35,36,37,38] that require extension and regular updates as commercial suppliers continually optimize printing materials. These optimizations, and also the market introduction of new formulations, aim to improve printing outcomes, simplicity and speed of printing, mechanical properties, and aesthetic quality—such as surface finish and visual appearance of printed parts—opening the field for new applications while keeping pace with rapidly evolving hardware.

This results in an ever-wider variety of materials based on the same base polymers, which are typically marketed under the same polymer designation. However, they differ significantly in the applications for which they are optimized and through polymer modifications, blends, and additives, all of which have the potential to substantially alter X-ray interaction properties. Thus, these new (optimized) printing materials need to be characterized with regard to x-ray interaction properties, including energy dependence of attenuation, since alterations in the base polymer matrix as well as in fillers and additives will impact both, and thus be relevant for material usability in radiologic phantoms.

This results in an ever-widening variety of materials based on the same base polymers, typically marketed under identical polymer designations. However, these variants differ substantially in targeted applications, polymer modifications, blends, and additives, all of which can markedly alter X-ray interaction properties. Consequently, attenuation and its energy dependence must be characterized to determine radiological properties and material suitability for radiographic phantoms, and data must be updated regularly. Since manufacturers may retain the same product name for reformulated printing polymers, changes in X-ray interaction properties can also occur between production batches.

This work updates the 2021 dataset on X-ray attenuation properties of 3D printing materials at diagnostic energies [32], evaluating a broad selection of currently available products and comparing them to prior results. It accounts for the substantial expansion in photopolymerization printing (SLA/DLP) options since then, including both affordable consumer-grade printers and the wide variety of compatible printing resins, focusing on accessible consumer-grade products suitable for any research laboratory. For FDM printing filaments, the study concentrates on the most commonly used base polymer systems (PLA, polymers with styrene backbone as ABS and HIPS, and styrene-free polyesters as PETG-/PCTG-based filaments) and excludes polymers with organic fillers (like wood- and cork-filled PLAs) or fibre-reinforced composites.

## 2. Materials and Methods

### 2.1. Filaments, Resins, and 3D Printers

Table 1 lists the filaments used in this study. Two fused deposition modelling (FDM) printers were employed: a Creality K2 Plus (Shenzhen Creality 3D Technology Co., Ltd., Shenzhen, China) and an FLSUN V400 (Zhengzhou Chaokuo Electronic Technology Co., Ltd., Zhengzhou, China). The Creality system features an actively heated enclosed build chamber, while the FLSUN printer, lacking an enclosure, allows continuous visual inspection of the build process for real-time adjustment and troubleshooting. Printing parameters were independently optimized for each filament to maximize surface quality and mass density, minimizing void formation within the printed structures. Mass density of the samples was compared to filament mass densities provided in the technical data sheets of the filaments to ensure maximum density was achieved. Compared to the previous study, clear improvements in both filaments and printing hardware were observed, as optimization of printing parameters required significantly fewer prints with adapted settings per filament type. All but one ABS filament achieved the optimum (maximum) density with a flow rate of 1 (100%), and in no case was a layer height below 0.15 mm necessary.

The selection of printing resins (Table 2) was designed to represent the major resin classes, ranging from hobby-grade water-washable formulations and resins optimized for visual aesthetics to technical photocurable resins for specialized rapid-prototyping applications, including tough materials capable of withstanding substantial mechanical stress and high-temperature-resistant model resins. All resins included in this study were commercially available and compatible with a wide range of printers and not restricted to any proprietary printing system. Samples were printed using DLP desktop printers (Elegoo Mars 3 Pro and Mars 4 Ultra, Shen-zhen Elegoo Technology Co., Ltd., Shen-zhen, China).

Printing parameters were determined for each resin individually before printing the samples using the Photonsters Validation Matrix v2 [39]. After printing, samples were cleaned using IPA (Iso Propyl Alcohol) and/or water, washed in deionized water, dried, and post-cured in a dedicated curing station (Wash & Cure, Anycubic, Shenzhen Anycubic Technology Co., Ltd., Shen-zhen, China).

### 2.2. Sample Evaluation

Cylindrical samples 2 cm in diameter and 3 cm in height were printed for HU value measurements in the CT scanner. Mass densities were assessed by weighing the specimens on a self-calibrating analytical balance (Sartorius AG, Göttingen, Germany) and determining their geometric dimensions with a digital calliper (Mitutoyo, Kawasaki, Japan). Each specimen’s height was measured three times at distinct positions, and diameters were determined at the top, middle, and bottom levels in two perpendicular orientations. Mean values of these measurements were used to calculate the volume to derive mass densities.

**Figure 1:**
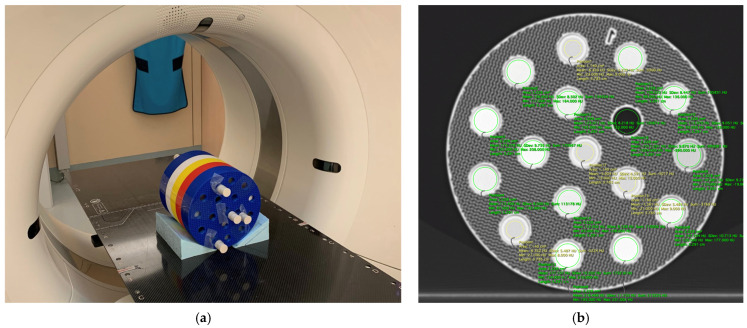
(**a**) CT phantom holding the printed samples positioned in the CT scanner gantry. PMMA tubes with plugs contain demineralized water. (**b**) Sample slice of Section 1 indicating ROI placement to determine HU values. One position in this section is empty and is used to check the HU value measured in air.

For CT scanning, samples were embedded into a cylindrical phantom of 20 cm diameter comprising 5 sections (Figure 1a), each accommodating up to 17 samples, one at the central position and 5 and 11 positions arranged in 2 concentric rings, respectively (Figure 1b). Four of these positions (one central, one in the inner ring, and two in the outer ring) were used to hold PMMA tubes filled with demineralized water. This enabled precise normalization of the HU values measured for the samples to exactly zero (representing water) by subtracting the corresponding water value measured in the respective ring in the matching section. The phantom was 3D-printed using Elegoo Rapid PLA+ in various colours and an infill density of 30% with a gyroid infill pattern on the FLSUN V400.

CT scanning was performed in a Siemens Somatom AS (Siemens Healthineers, Erlangen, Germany). Scans were conducted at 70, 80, 100, 120, and 140 kV using a modified abdomen protocol. A total collimation of 19.2 mm (32 × 0.6 mm), 1 mm reconstructed slice thickness, and the I40s3 reconstruction kernel were used. To allow higher effective mAs values to minimize image noise, a pitch of 0.55 was selected. For 70 and 80 kV, 900 and 1100 mAs yielding CTDI_vol_ values of 13.8 mGy and 27.05 mGy, respectively, were set; for 100, 120, and 140 kV, mAs values of 1037, 1000, and 850 mAs, yielding CTDIvol values of 50 mGy, 82.93 mGy, and 101.43 mGy, respectively, were applied. mAs values were selected at every kV setting to represent the best compromise between the highest possible dose to obtain very low noise images for best accuracy in HU determination and the technical limitations of the tube. HU values were measured using the Horos medical image viewing and reporting software package (version 3.3.6) available under the GNU Lesser General Public License 3.0 (Horosproject.org, sponsored by Nimble Co LLC Purview, Annapolis, MD, USA). Circular ROIs were placed within the samples, slightly smaller than the sample diameter and length, to avoid partial volume artifacts (cf. Figure 1b). The standard deviations of the average HU values from individual measurements in the 1 mm thick slices within each sample served as measures of measurement uncertainty for the HU values. After removal of the topmost and bottom slices due to partial volume averaging artefacts, typically 26 slices and thus 26 individual HU measurements were available to derive the STD values.

## 3. Results

Table 3 shows the mass densities of the printed FDM samples. For 15 of the printed samples, the measured mass densities agreed with the manufacturer’s specifications after optimization of printing parameters from the filament data sheets within their respective measurement uncertainties (maximum ±0.01 g/cm^3^, determined from mass and volume measurements).

In all three HIPS filaments, the achievable density was lower than manufacturer specified filament density (Δρ of 0.06 to 0.02 g/cm^3^, both ±0.01 g/cm^3^), as also observed in the preceding study. Similar differences were observed for ABS and one PLA filament (Δρ = 0.02 ± 0.01 g/cm^3^), whereas the densities of Bambu Lab PETG HF and Sunlu PVB slightly exceeded the nominal values (Δρ = +0.02 ± 0.01 g/cm^3^).

Densities of the cured resin samples printed with DLP/SLA are listed in Table 4. The cured resins exhibited densities between 1.18 g/cm^3^ and 1.42 g/cm^3^, with 27 of the 29 cured resins falling between 1.18 g/cm^3^ and 1.25 g/cm^3^.

**Figure 2:**
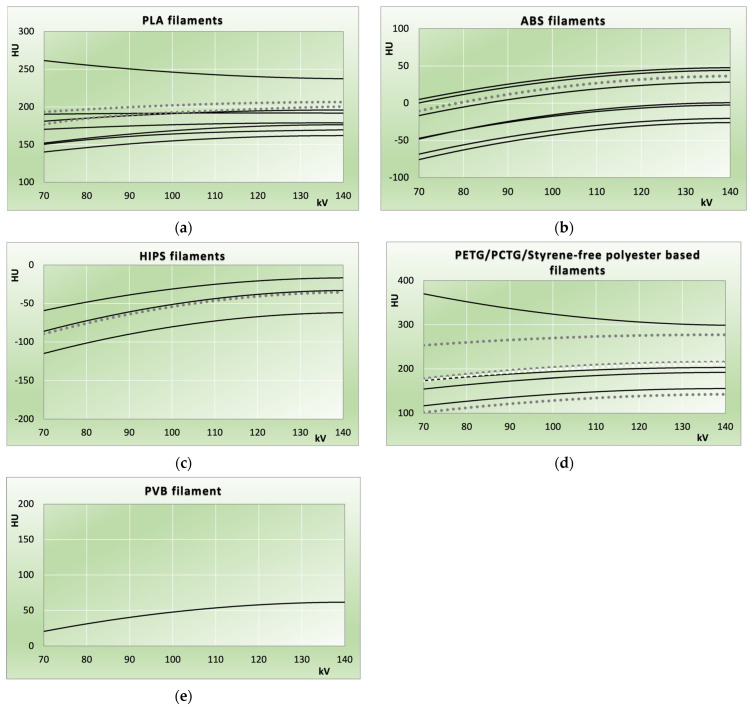
HU values measured in the samples printed from thermoplastic filaments using FDM. Solid lines and the dashed line represent current measurements, and dotted lines provide a comparison to the 2021 study. (**a**) PLA-based filaments; (**b**) ABS-based; (**c**) HIPS; (**d**) polymer filaments based on styrene-free polyesters from the PET/PETG/PCTG family; and (**e**) PVB.

**Figure 3:**
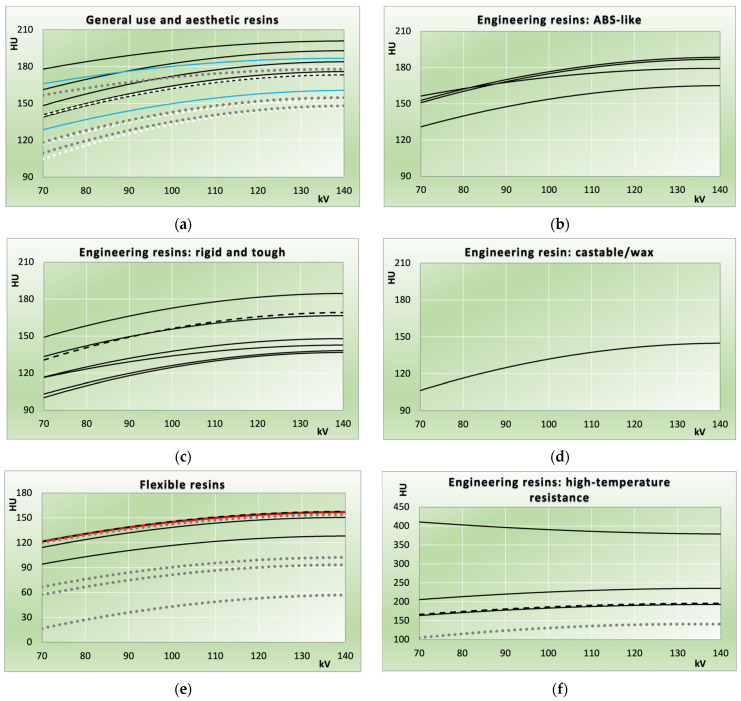
HU values of photocurable resin samples vs. tube potential. Solid and dashed lines: measurements; dotted lines: comparison to 2021 study. (**a**): Resins intended for general and aesthetic prints (black: IPA washable resins; blue: water-washable); (**b**) resins with mechanical features similar to ABS; (**c**) rigid and tough engineering resins; and (**d**) a castable waxlike resin. For better comparison, the HU axis is similar in (**a**–**d**); (**e**) flexible resins. Note: Prima Creator Flex is shown in red since, otherwise, it would not be distinguishable from 3DMaterials Superfast Superflex. (**f**) Resins with high temperature resistance.

Figure 2 and Figure 3 illustrate HU value measurements versus beam hardness. Data from the measured samples (solid and dashed lines) is compared to the results from the 2021 study [32] (dotted lines). Dashed lines are used when measurements from different samples are nearly identical, rendering the corresponding solid lines indistinguishable or not clearly assignable. In Figure 3e, red colouring is used to distinguish resins that are otherwise indiscernible. If this applies to the dotted lines representing data from the 2021 study, white colouring is used instead of grey (Figure 2d and Figure 3a).

Uncertainty indicators from standard deviations of slice-averaged HU values are not shown in the figures, as these were <2 HU on average (<3 HU in all but two samples: Bambu Lab PETG HF red at 3.7 HU average, and FormFutura Easyfil HIPS at 15.8 HU). Lines correspond to second-order polynomial regressions of HU values versus kV, with coefficients of determination (R^2^) averaging 0.99 (range 0.89–1.00) for FDM samples and 1.00 (range 0.99–1.00) for photocured resin samples. In both figures, individual filaments (Figure 2) and photocured resins (Figure 3) can be identified from their order in Table 1 and Table 2, or Table 3 and Table 4, respectively. In these tables, polymers or resins are arranged from highest to lowest attenuation at 120 kV used as reference tube potential, corresponding to top-to-bottom order in the figures.

PLA-based filaments ranged from 124 to 384 HU (120 kV, Figure 2a). The ABS filaments used in this study covered HU values from −32 to 43 HU (120 kV, Figure 2b). All HIPS filaments exhibited negative HU values across the entire kV range, with the three investigated variants ranging from −69 to −22 HU (120 kV); eSUN HIPS natural, in between the other two, exhibited nearly identical HU values compared to the one measured in the 2021 publication. As observed for all other filament types, HIPS also displayed a range of X-ray attenuations, but entirely within the negative HU region best corresponding to adipose tissue.

All PETG-/PCTG-based filaments fell between 152 and 308 HU (120 kV), indicating attenuation above the soft tissue range (cf. Figure 2d). The only filament filling the gap in the low positive HU range identified was PVB (57 HU at 120 kV).

Figure 3 compares photocurable resin printing samples with those from the previous study. In panel (a), regular IPA (isopropyl alcohol)-washable resins are shown in black and water-washable resins in blue; HU values (120 kV) ranged from 157 to 198 HU.

Technical resins with enhanced mechanical properties are found in the “ABS like” category (cf. Figure 3b, where the name indicates similar mechanical properties, and in the resins marketed as rigid and tough (Figure 3c). The former ranged from 161 to 185 HU (120 kV) with the Sunlu 14k solid grey resin exhibiting a flatter energy dependence compared to the others. Rigid and tough resins were available from 133 to 181 HU (120 kV).

One castable/wax-like resin exhibited 141 HU (120 kV). Flexible resins (Figure 3e) ranged from 125 to 154 HU (120 kV), with the three highest values (3D Materials SuperFast SuperFlex: 146 HU; PrimaCreator Flex: 145 HU; 3D Materials SuperFast SuperElastic: 139 HU at 120 kV) nearly identical to Flexible Resin Clear (PhotoCentric3D) from the previous study. In Figure 3e, 3D Materials SuperFast SuperFlex is shown using a dashed line to distinguish it from PrimaCreator Flex (red).

High-temperature-resistant resins (Figure 3f) showed elevated attenuation close to and exceeding 200 HU at 120 kV, attributable to additives increasing thermal stability. HU values at 120 kV ranged from 190 to 384 HU. Phrozen TR250LV (HDT 80–120 °C), Siraya Tech Sculpt Clear (HDT ≤ 180 °C), and Phrozen TR300 (HDT 160 °C) exhibited lower HU values with increasing HU for harder spectra, identical to all other resins tested. Conversely, Siraya Tech Sculpt Ultra White’s high filler loading resulted in the highest attenuation in combination with decreasing HU with increasing kV—indicative of higher-Z additives.

## 4. Discussion

SLA/DLP resins spanned a broad range of moderate positive HU values, while FDM filaments covered negative to moderate positive HU across PLA, ABS, PETG/PCTG, and HIPS variants, with energy dependence in most cases indicating lower effective atomic numbers than water. Compared to the 2021 study, materials now offer a wider choice and variability of attenuations and finer nuances.

It is still challenging to find printing materials with appropriate X-ray interaction properties simulating human tissues in radiographic phantoms [40]. In the soft tissue-equivalent range (low-attenuating filaments or SLA/DLP resins), energy dependence yields lower HU values at softer spectra due to an insufficiently low effective atomic number to directly simulate water or water-dominated soft tissues. Relative attenuation differences for anthropomorphic contrasts can, nevertheless, be realized over a wide range by selecting the most appropriate materials, including across spectra. Simulation of adipose tissues at negative HU values is easier [33], as their reduced atomic number yields an energy dependence easier to mimic with 3D printing materials. Whereas no commonly available photocurable resin system examined offers sufficiently low attenuation, HIPS and some ABS filaments provide suitable X-ray radiodensities. To reduce attenuation, controlled underfilling via reduced flow rates [41,42] or appropriate infill patterns [43,44] can be applied. Dithering and using multiple resins in one print also allow fine-tuning to anthropomorphic contrasts and attenuations with very high spatial resolution, but require printer modification and industrial 3D printers [45]. To expand the range of X-ray attenuation properties in SLA/DLP printed phantoms, resins can be mixed. This is commonly done to optimize mechanical properties (e.g., by adding a low percentage of flexible resin to standard or rigid resins). Another approach is doping resins with higher atomic number additives to correct the typically too-low effective atomic number or adding contrast media like iodine to simulate post-contrast tissue uptake [46].

Reporting X-ray attenuation properties of 3D printing materials beyond mass attenuation coefficients μ/ρ, such as linear attenuation coefficients or HU, requires specification of sample mass density. For FDM filaments, printing parameters substantially influence material density [32], which frequently deviates from manufacturer specifications; consequently, inter-study HU comparisons are unreliable without density reporting. For SLA/DLP photocurable resins, cured density remains independent of printing parameters but is sensitive to post-curing; manufacturer-provided values, when available, normally pertain to uncured resin and are thus inapplicable.

### Comparison with Previous Study

PLA filaments exhibited wider variation in X-ray attenuations than in the previous study, with some also showing flatter energy dependence in the range where the prior work reported PLA HU values (e.g., eSUN PLA+ HS, black; dashed line in Figure 2a). Among ABS-based filaments, the three with the highest X-ray attenuation most closely match the material from the 2021 study (transparent ABS, Verbatim GmbH, Eschborn, Germany), which was likely closer to the base acrylonitrile–butadiene–styrene polymer than modern printing filaments. Modern developments feature substantially reduced warping during printing and markedly higher achievable print speeds.

While styrene-free polyesters (PET-/PETG-based) in the 2021 study showed substantial HU variability, non-glycol-modified PET plays a lesser role in modern formulations, whereas PETG appears in many variations. The filament with the highest X-ray attenuation in this class (Bambu Lab PETG HF, red) shows a deviant energy dependence, decreasing HU with harder spectra, indicating an additive or colourant with a higher atomic number responsible for the elevated attenuation.

In SLR/DLP printing, due to the substantially expanded resin selection, not all resin classes are directly comparable to the previous study. Valid comparisons exist for general use and aesthetic resins (Figure 3a), flexible resins (Figure 3e), and high-temperature engineering resins (Figure 3f), though only one such resin was available in the prior work. In Figure 3a, general-purpose/aesthetic resins are divided into standard (to be cleaned with alcohol/IPA after printing) and their water-washable counterparts. A representative of the latter, Elegoo 8K water-washable resin in space grey, exhibited lower attenuation (120 kV) than its standard IPA-washable version in combination with a flatter energy dependence, most closely resembling the highest-attenuating resin from the 2021 study. Meanwhile, four resin systems in the previous study fell below or near 150 HU at 120 kV (blue and white dotted lines); the lowest general-use resin here (PrimaCreator Water Washable Clear) showed higher attenuation in direct comparison.

Flexible resins generally exhibited higher X-ray attenuation than those in the previous study, though the resins with the highest attenuation in this study very closely matched PhotoCentric3D Flexible Resin Clear from the 2021 study. The flexible resin, having exhibited the lowest attenuation in the 2021 study, Formlabs Flexible 80A (Formlabs Inc., Somerville, MA, USA), is still available, and a V2 formulation has been added with Shore 50A, but was excluded owing to Formlabs’ proprietary ecosystem. However, its elastomeric properties and low attenuation nonetheless may provide potential for specialized soft-tissue phantom applications. Ongoing developments in printing resins since the last study have resulted in improved mechanical reliability or printability in consumer-grade SLA/DLP systems, emphasizing the need to validate commercial materials for phantom applications.

## 5. Limitations

This study examined a selection of currently available commercial 3D printing materials for FDM filaments and SLA/DLP resins, but it cannot encompass the full market diversity, which continues to evolve rapidly. Notably, within FDM filament product lines, different colours incorporate distinct colourants and additives—often including mineral fillers—that result in varying effective atomic numbers influencing X-ray attenuation properties and energy dependencies. The impact of these colour-specific variations was not systematically quantified here and warrants dedicated follow-up investigations. CT measurements were conducted on a single scanner using standard reconstruction parameters; inter-scanner and algorithm-related differences in HU values, although expected to be minor relative to material-inherent variations, were not considered.

## 6. Conclusions

Base polymer designations or usage class alone do not adequately characterize X-ray interaction and attenuation properties or indicate which tissues can be mimicked in radiological phantoms using the corresponding printing polymer. Commercially available 3D printing materials now span a wider range of X-ray attenuations with finer gradations, enabling replication of clinically relevant tissues and contrasts for patient- and task-specific phantoms. Compared to the 2021 study, FDM filaments (PLA, ABS, and HIPS) show greater variability, PETG-family copolymers remain inhomogeneous, and SLA/DLP resins offer expanded options for 3D-printed phantoms. Data presented facilitates material selection for radiographic phantoms while underscoring the need for ongoing characterization of evolving material formulations.

## Figures and Tables

**Table 1:** Filaments used in this study for fused deposition modelling (FDM) printers are based on polymer or application class. Filaments are sorted within groups from highest to lowest HU value at 120 kV.

Base Polymer/Application Class	Manufacturer	Filament Type and Colour
PLA (Polylactic acid)-based filaments		
	FormFutura (Formfutura BV, Nijmegen, The Netherlands)	Volcano PLA, gold (technical filament)
	Elegoo (Shen-zhen Elegoo Technology Co., Ltd., Shen-zhen, China)	PLA Pro, light blue
	eSUN (Shenzhen Esun Industrial Co., Ltd., Shen-zhen, China)	PLA+ HS, black
	Creality (Shenzhen Creality 3D Technology Co, Ltd., Shen-zhen, China)	Hyper PLA, white
	Fiberlogy (Fiberlogy SA, Brzezie, Poland)	HS PLA Clear, pure transparent
	Elegoo	Rapid PLA+, red
	Fiberlogy	Impact PLA, orange (technical filament)
HIPS (high-impact Polystyrene)-based filaments		
	Fillamentum (Fillamentum Manufacturing Czech s.r.o., Hulín, Czech Republic)	HIPS Extrafill, sky blue
	eSUN	HIPS, natural
	FormFutura	EasyFil HIPS, natural
ABS (Acrylonitrile butadiene styrene)-based filaments		
	Fiberlogy	Easy ABS, pure transparent
	3DJake (Niceshops GmbH, Paldau, Austria)	Nice ABS, red
	Fillamentum	ABS Extrafill, transparent
	FormFutura	TitanX, natural
	Creality	Hyper ABS, grey
	eSUN	ABS+ HS, red
	Fillamentum	ABS Extrafill, natural
Styrene-free polyester-based filaments: PET (Polyethylene terephthalate)-, PETG (Glycol modified PET)-, and PCTG (Polycyclohexylenedimethylene terephthalate)-based filaments		
	BambuLab (Bambulab Ltd., Kowloon, Hong Kong, China)	PETG HF, red
	Creality	Hyper PETG, transparent
	BambuLab	PETG Translucent, teal
	3DJake	PCTG, transparent blue
PVB (Polyvinyl butyral)-based filament		
	Sunlu (Zhuhai Sunlu Industrial Co., Ltd., Zhongshan, China)	PVB, transparent

**Table 2:** List of printing resins for DLP/SLA. Resins are sorted within groups from the highest to lowest HU value of the printed and post-cured samples at 120 kV.

Application Class	Manufacturer	Type
General use and aesthetic resins		
	Elegoo	8k Standard resin, space grey
	3DMaterials (3DMaterials Co., Ltd., Anyang, Republic of Korea)	Superfast 8k, white
	Elegoo	8k Water washable, space grey
	Siraya Tech (Siraya Tech Corp., San Gabriel, CA, USA)	Craft, ultra clear
	Siraya Tech	Built, sonic grey
	3DMaterials	Superfast 8k, clear
	PrimaCreator (Prima Printer Nordic AB, Malmö, Sweden)	Water washable, clear
ABS-like engineering resins		
	Siraya Tech	Fast ABS-like resin, grey
	Siraya Tech	Fast ABS-like resin, tough grey
	Sunlu	14k ABS-like Resin, solid grey
	Elegoo	ABS-Like resin 3.0, translucent
Tough and rigid engineering resins		
	3DMaterials	Super PCS, grey
	3DMaterials	Super PP, clear
	Siraya Tech	Blue Clear V2
	Siraya Tech	BLU Nylon, black
	PrimaCreator	Tough, white
	PrimaCreator	Tough, clear
	Phrozen (Phrozen Tech Co. Ltd., Hsinchu City, Taiwan, China)	Onyx Pro 410, black
Flexible resins		
	3DMaterials	Superfast Superflex, clear
	Prima Creator	Flex, clear
	3DMaterials	Superfast Superelastic, clear
	FormFutura	Engineering LCD Resins Flex 63A
High-temperature-resistant engineering resins		
	Siraya Tech	Scuplt Ultra, white
	Phrozen	TR300, grey
	Siraya Tech	Sculpt, clear
	Phrozen	TR250LV, grey
Castable resins		
	Liqcreate (ALT Group BV, Utrecht, The Netherlands)	Wax Castable, blue

**Table 3:** Mass densities of printed FDM samples compared to filament density (nominal, according to manufacturer specifications). Values in italics indicate measured sample density not within nominal filament density as indicated in the technical data sheet of filament or manufacturer specification.

Base Polymer/Application Class	Filament/Sample	Sample Density [g/cm^3^]	Nominal Filament Density [g/cm^3^]
PLA-based filaments			
	FormFutura Volcano PLA, gold	1.27 ± 0.01	1.27
	Elegoo PLA Pro, light blue	1.24 ± 0.01	1.24
	eSUN PLA+ HS, black	1.23 ± 0.01	1.24
	Creality Hyper PLA, white	*1.23 ± 0.01*	*1.25*
	Fiberlogy HS PLA Clear, pure transparent	1.24 ± 0.01	1.24
	Elegoo Rapid PLA+, red	1.22 ± 0.01	1.23
	Fiberlogy Impact PLA, orange	1.21 ± 0.01	1.22
HIPS-based filaments			
	Fillamentum HIPS Extrafill, sky blue	*1.03 ± 0.01*	*1.05*
	eSUN HIPS, natural	*1.01 ± 0.01*	*1.05*
	FormFutura EasyFil HIPS, natural	*0.99 ± 0.01*	*1.05*
ABS based filaments			
	Fiberlogy Easy ABS, pure transparent	1.10 ± 0.01	1.09
	3DJake Nice ABS, red	1.11 ± 0.01	1.10
	Fillamentum ABS Extrafill, transparent	1.08 ± 0.005	1.08
	FormFutura TitanX, natural	1.05 ± 0.01	1.05
	Creality Hyper ABS, grey	1.05 ± 0.01	1.04
	eSUN ABS+ HS, red	1.03 ± 0.01	1.04
	Fillamentum ABS Extrafill, natural	*1.02 ± 0.005*	*1.04*
Styrene-free polyester-based filaments			
	BambuLab PETG HF, red	*1.30 ± 0.01*	*1.28*
	Creality Hyper PETG, transparent	1.28 ± 0.01	1.27
	BambuLab PETG Translucent, teal	1.26 ± 0.01	1.25
	3DJake PCTG, transparent blue	1.22 ± 0.01	1.23
PVB filament			
	Sunlu PVB, transparent	*1.10 ± 0.01*	*1.08*

**Table 4:** Measured densities of photopolymer samples. Densities for printed and post-cured samples are not specified by the manufacturer for comparison and thus are not provided.

Application Class	Resin/Sample	Sample Density [g/cm^3^]
General use and aesthetic resins		
	Elegoo 8k Standard resin, space grey	1.25 ± 0.01
	3DMaterials Superfast 8k, white	1.25 ± 0.01
	Elegoo 8k Water washable, space grey	1.23 ± 0.01
	Siraya Tech Craft, ultra clear	1.24 ± 0.01
	Siraya Tech Built, sonic grey	1.23 ± 0.01
	3DMaterials Superfast 8k, clear	1.23 ± 0.01
	PrimaCreator Water washable, clear	1.21 ± 0.01
ABS-like engineering resins		
	Siraya Tech Fast ABS-like resin, grey	1.25 ± 0.01
	Siraya Tech Fast ABS-like resin, tough grey	1.25 ± 0.01
	Sunlu 14k ABS-like Resin, solid grey	1.22 ± 0.01
	Elegoo ABS-Like resin 3.0, translucent	1.22 ± 0.01
Tough and rigid engineering resins		
	3DMaterials Super PCS, grey	1.25 ± 0.01
	3DMaterials Super PP, clear	1.23 ± 0.01
	Siraya Tech Blue Clear V2	1.22 ± 0.01
	Siraya Tech BLU Nylon, black	1.20 ± 0.01
	PrimaCreator Tough, white	1.19 ± 0.01
	PrimaCreator Tough, clear	1.18 ± 0.01
	Phrozen Onyx Pro 410, black	1.19 ± 0.01
Flexible resins		
	3DMaterials Superfast Superflex, clear	1.22 ± 0.01
	Prima Creator Flex, clear	1.21 ± 0.01
	3DMaterials Superfast Superelastic, clear	1.23 ± 0.01
	FormFutura Engineering LCD Resins Flex 63A	1.19 ± 0.01
High-temperature-resistant engineering resins		
	Siraya Tech Sculpt Ultra white	1.42 ± 0.01
	Phrozen TR300, grey	1.30 ± 0.01
	Siraya Tech Sculpt clear	1.25 ± 0.01
	Phrozen TR250LV, grey	1.25 ± 0.01
Castable resins		
	Liqcreate Wax Castable, blue	1.20 ± 0.01

## Data Availability

Data are contained within the article.
